# Pre-emptive pharmacological inhibition of fatty acid–binding protein 4 attenuates kidney fibrosis by reprogramming tubular lipid metabolism

**DOI:** 10.1038/s41419-021-03850-1

**Published:** 2021-06-03

**Authors:** Yuting Chen, Yue Dai, Kaixin Song, Yi Huang, Le Zhang, Cuntai Zhang, Qi Yan, Hongyu Gao

**Affiliations:** Department of Geriatrics, Tongji Hospital, Tongji Medical College, Huazhong University of Science and Technology, Wuhan, China

**Keywords:** Metabolism, Chronic kidney disease

## Abstract

Kidney fibrosis is a hallmark of chronic kidney disease (CKD) progression that is caused by tubular injury and dysregulated lipid metabolism. Genetic abolition fatty acid-binding protein 4 (FABP4), a key lipid transporter, has been reported to suppress kidney interstitial fibrosis. However, the role and underlying mechanism of chemical inhibition of FABP4 in fibrotic kidney have not been well-documented. Here, we examined preemptive the effect of a FABP4 inhibitor, BMS309403, on lipid metabolism of tubular epithelial cells (TECs) and progression of kidney fibrosis. The expression of FABP4 was significantly elevated, concomitated with the accumulation of lipid droplets in TECs during kidney fibrosis. Treatment with BMS309403 alleviated lipid deposition of TECs, as well as interstitial fibrotic responses both in unilateral ureteral obstruction (UUO)-engaged mice and TGF-β-induced TECs. Moreover, BMS309403 administration enhanced fatty acid oxidation (FAO) in TECs by regulating peroxisome proliferator-activated receptor γ (PPARγ) and restoring FAO-related enzyme activities; In addition, BMS309403 markedly reduced cell lipotoxicity, such as endoplasmic reticulum (ER) stress and apoptosis in fibrotic kidney. Taken together, our results suggest that preemptive pharmacological inhibition of FABP4 by BMS309403 rebalances abnormal lipid metabolism in TECs and attenuates the progression of kidney fibrosis, thus may hold therapeutic potential for the treatment of fibrotic kidney diseases.

## Introduction

Chronic kidney disease (CKD) is recognized as a global public health concern, directly promoting the loss of kidney function. Around 10% of the general population are affected by different types of kidney disorders. Kidney fibrosis is one of the main pathological features of CKD and is closely associated with kidney function. Despite the current burden of fibrosis-related human disease, there are currently few specific treatments for fibrosis^1^. Therefore, investigated the mechanism of kidney fibrosis is of great significance to the treatment of CKD.

The energy supply to the healthy kidney predominantly originates from the oxidization of fatty acids due to mitochondria are abundantly present in tubular epithelial cells (TECs). Under normal conditions, the lipid metabolism of TECs is tightly controlled to maintain the energy diamond of the kidney^2^; whereas during kidney fibrogenesis, lipid droplets accumulation in the kidney due to dysregulated lipid metabolism (e.g., defective fatty acid oxidation [FAO] or excess lipid uptake), will lead to cytotoxicity termed as “lipotoxicity”, which causes oxidative stress, endoplasmic reticulum (ER) stress, inflammatory infiltration, apoptotic cell death, and ultimately kidney failure^3–6^. PPARalpha agonist fenofibrate attenuated kidney tubular injury and collagen deposition in folic acid and UUO-induced kidney fibrosis models^2^. Overexpression of peroxisome proliferator-activated receptor γ (PPARγ) coactivator-1a (PGC1α) in TECs also ameliorated folic acid-induced kidney fibrosis and cell apoptosis^2^. Moreover, our previous study revealed that inhibition of fatty acid transport protein 2 (FATP2) by lipofermata attenuated kidney lesion and improved collagen deposition^7^. Therefore, regulating lipid metabolism may be an effective therapeutic target for CKD treatment.

Fatty acid-binding protein 4 (FABP4, also known as aP2) is a member of FABPs family which is highly expressed in adipocytes^8–10^ and macrophages^11–13^. It is well established that the expression of FABP4 could be regulated by PPARγ agonists, insulin, and fatty acids^9,10,14^. In recent years, more attention has been paid to the role of FABP4 in the development of kidney disease. Previous studies have shown FABP4 is expressed in multiple parts of the kidney, such as peritubular endothelial cells, glomerulus, mesangial cells, and tubular cells^15–18^. Inhibition of FABP4 significantly reduces inflammation infiltration, kidney function, and tubular damage in rhabdomyolysis-, cisplatin-induced acute kidney injury (AKI)^19,20^, and hyperuricemic nephropathy^21^. In addition, FABP4 is also reported to participate in the development of diabetic nephropathy (DN), knockdown FABP4 by siRNA suppresses cell apoptosis in high glucose-, oleic acid-, and palmitic acid-induced HMCs by inhibiting ER stress^16^. Furthermore, FABP4 knockout mice also show reduced kidney interstitial fibrosis than wild-type mice in the obstructive kidney^22,23^. However, whether chemical FABP4 inhibitor exerted a favorable renoprotective role against kidney interstitial fibrosis and the involved mechanisms remained unknown.

BMS309403 is a synthetic small molecule FABP4 inhibitor which has been demonstrated to prevent and treat type 2 diabetes and atherosclerosis^24^. Treatment genetically obese mice with BMS309403 ameliorates glucose homeostasis and insulin sensitivity, reduces adipose tissue inflammation, and lipid deposition in the liver^24^. BMS309403 administration also significantly limits atherosclerotic lesion formation and improves endothelial function in *ApoE*^*−/*−^ mice^24,25^, attenuates acute liver injury and high-fat high-cholesterol diet-induced non-alcoholic fatty liver disease^26^. In this study, we evaluated the therapeutic effect of BMS309403 on the progression of fibrotic responses both in vivo and in vitro models. Furthermore, we investigated the effect of BMS309403 on the tubular lipid metabolism as well as cell lipotoxicity associated with the development of kidney fibrosis.

## Materials and Methods

### Antibodies and reagents

We used antibodies to FABP4 (Abcam, ab92501, WB 1:1000 dilution; IF 1:50 dilution), fibronectin (Abcam, ab45688, 1:5000 dilution), α-SMA (Abcam, ab124964, 1:10,000 dilution), collagen-I (Proteintech, 14695-1-AP, 1:1000 dilution), TGF-β1 (Proteintech, 21898-1-AP, 1:1000 dilution), p-Smad2 (CST, 3104 S, 1:1000 dilution), p-Smad3 (CST, 9520 T, 1:1000 dilution), PGC1α (Abcam, ab54481, 1:1000 dilution), PPARγ (Proteintech, 16643-1-AP, 1:1000 dilution), caspase-3 (Proteintech, 19677-1-AP, 1:1000 dilution), Bcl-2 (Santa Cruz, sc-7382, 1:1000 dilution), BIP (CST, 3177 S, 1:1000 dilution), CHOP (CST, 2895 T, 1:1000 dilution). Recombinant human TGF-β1 (Peprotech, 100-21C-10), Oil Red O (Sigma-Aldrich, O0625). FABP4 inhibitor BMS309403 (R&D systems, 5258/10).

### Animals

All procedures were conducted in accordance with the guidelines of the National Health and Medical Research Council of China and were approved by the animal ethics review board of Tongji hospital of Tongji Medical College. Male C57BL/6 mice (9–10 weeks old; six per experimental group) received an operation with left ureteral obstruction below the kidney pelvis and were treated with BMS309403 (40 mg/kg/day) in flaxseed oil at 1 h prior to surgery and continuously received daily for UUO duration. Controls received a vehicle consisted of flaxseed oil alone. The mice were sacrificed on day 7 after surgery, and the kidneys were obtained for further analysis.

### Assessment of biological parameters

The serum and kidney tissue levels of triglyceride (TG), cholesterol (TC), and free fatty acid (FFA) of mice were detected using commercial kits (Changchun huili, C061/C063; Jiubang, CK-E28753) according to the manufacturer’s instructions.

### Cell lines and culture conditions

The human proximal TEC line HK-2 was obtained from China Centre for Type Culture Collection (CCTCC, China). Cells were maintained in DMEM/F-12 (Hyclone) media, consisting of 10% FBS, 100 U/mL penicillin, and 100 U/mL streptomycin. They were maintained in a humidified environment with 5% CO_2_ at 37 °C. At 80% confluence, cells were treated with 10 ng/ml TGF-β with or without BMS309403 for 48 h.

### Immunofluorescence assay

For kidney tissues, paraffin-embedded mouse kidneys were sectioned, de-paraffinized, and subsequently blocked in PBS containing 0.5% Triton and 5% normal goat serum for 1 h. Antigen retrieval was performed using citrate buffer prior to incubation with primary antibodies. The next day, the samples were incubated with species-specific fluorogenic secondary antibodies (Abbkine, USA), DAPI for 1 h. Images were collected using confocal microscopy (Nikon C2, Japan). Exposure setting were unchanged throughout acquisition. Staining was analyzed using ImageJ software.

### Histological analysis

We used 4% paraformaldehyde-fixed, paraffin-embedded kidney sections stained with hematoxylin-eosin (HE), periodic acid-Schiff (PAS), and Masson’s trichrome staining (MTS) according to the manufacturer’s instructions. The kidney tubular damage score was based on tubular necrosis grade, cast formation, tubular dilation, and brush border loss, with scores corresponding to the following percentages of kidney tubular damage: 0, 0%; 1, ≤10%, 2, 11 to 25%; 3, 26 to 45%; 4, 46 to 75%; and 5, ≥76%. The extent of tubulointerstitial damage was determined in successive fields examined in the entire cortical and juxtamedullary areas, suitable for evaluation, in each specimen^27^. Damaged areas were defined visually in each ×200 field and quantified as a percent of the total area under examination using computer assisted image analysis^27^. The mean score of each sample was compared. The samples were examined using microscope (Mshot, China), and the images were analyzed using ImageJ software (National Institutes of Health, Bethesda, MD).

### Oil red O staining

We used optimal cutting temperature compound (OCT)-embedded frozen sections for Oil red O staining. Kidney tissues or cell coverslips were first washed in PBS and subsequently fixed with 4% paraformaldehyde for 20 min, washed three times in PBS, incubated in 60% isopropyl alcohol for 10 sec, followed by staining with freshly prepared 60% Oil Red O solution (100% solution: 0.5 g of Oil Red O dissolved in 100 mL of isopropylene) for 30 min and washing with PBS. The samples were counterstained with hematoxylin. The slides were visualized using a Mshot microscope (Mshot, China). Staining positive and LDs number were quantified using ImageJ software following standard protocols^28^.

### CCK8 assay

Cell viability assay was measured by Cell Counting Kit-8 (Bimake, China). HK-2 cells were cultured at 1 × 10^4^ cells per well in a 96-well plate, when reaching 80–90% confluence, cells were treated with BMS309403 for 48 h. BMS309403 were diluted to obtain different concentration gradients. Absorbance was detected at 450 nm after treatment with 10 μL of CCK8 reagent for 2 h. The experiments were performed with six replicated wells per sample, and the assays were conducted in triplicate.

### Real-time quantitative PCR

RNA was isolated from harvested cells and kidneys using Hipure Total RNA mini kit (R4111-03, Magen, China). One microgram RNA was reverse transcribed using the ReverTra Ace qPCR RT kit (FSQ-101, Toyobo, Japan), and qRT-PCR was run in the ABI Step One Plus system (Applied Biosystems, USA) machine using SYBR Green Master Mix and gene-specific primers. The data were normalized and analyzed using the ΔΔCt method. The primers used are listed in Supplementary Table 1.

### Western blot

Cells or kidney tissues were lysed in RIPA buffer in the presence of 1% PMSF and 1% protease inhibitor cocktail, and then separated by 10% SDS-PAGE under reducing condition. After electrophoresis, samples were transferred to PVDF membranes (Millipore, USA) and blocked in 5% skim milk for 1 h at room temperature. Subsequently, the membranes incubated with indicated primary antibodies and following secondary antibodies. UVP imaging system (UVP, USA) were used to scan the membranes and Quantity One analysis software was applied to quantify the band intensity of blotted proteins.

### TUNEL staining

TUNEL was performed in 3-μM-thick sections of paraffin-embedded tissue with the In Situ Death Detected Kit Fluorescein (Roche, Indianapolis, IN) according to the manufacturer’s instructions.

### Statistics

All data were expressed at means ± SEM. Statistical analysis was performed using GraphPad Prism 8 (GraphPad Software Inc. San Diego, CA). Statistical analysis was performed with student’s *t*-test. One-way analysis of variance was used for multiple comparisons. *P* values <0.05 were considered to indicate a statistically significant difference.

## Results

### FABP4 is upregulated during kidney fibrosis

**Fig. 1:**
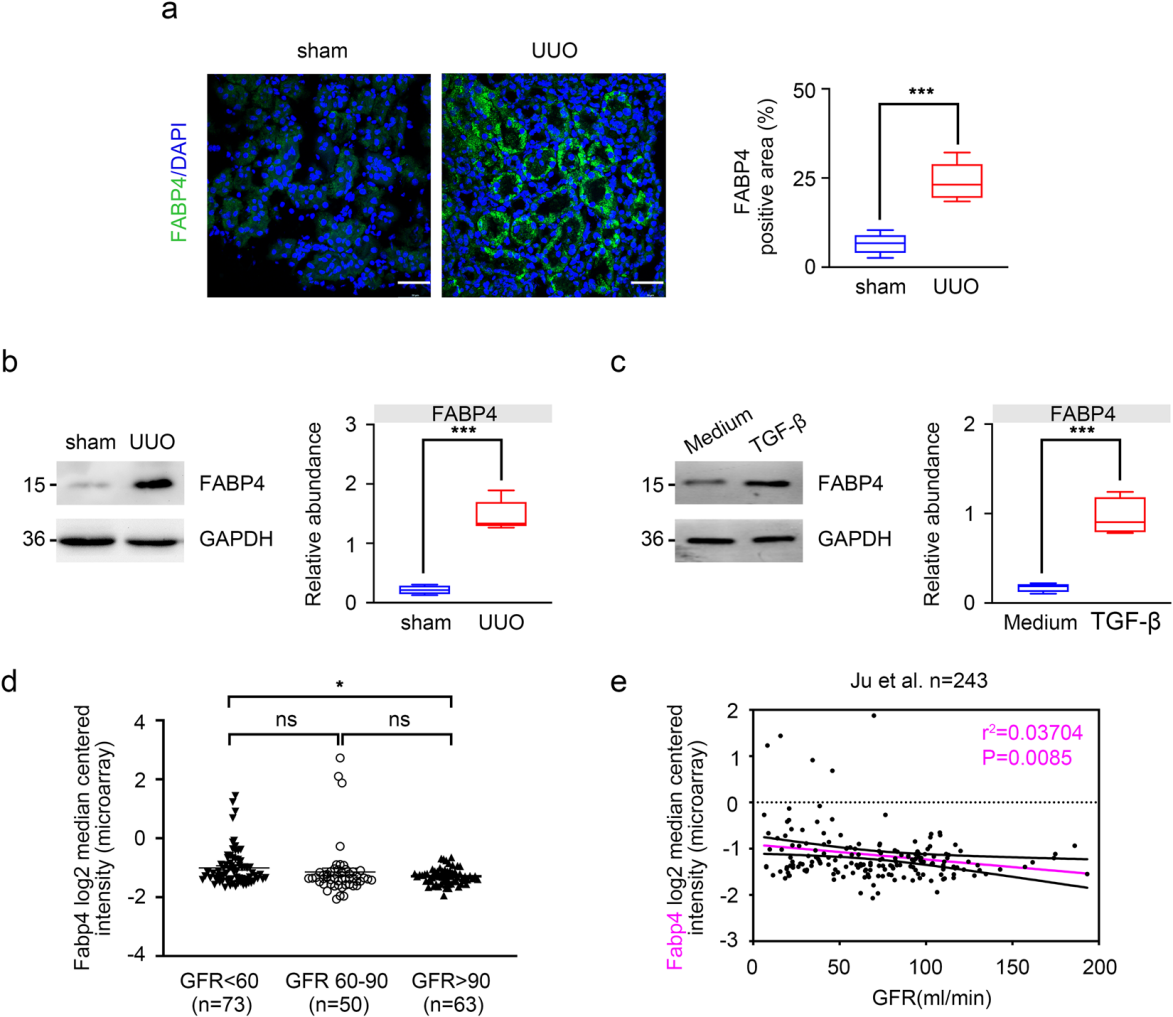
Expression of FABP4 in UUO mice and TGF-β-treated HK-2 cells. **a** Fluorescence microscopy revealed that FABP4 was mainly expressed in kidney tubular epithelial, and increased in UUO-treated mice. Quantitative image to depict fluorescence intensity (Right panel). Scale bars: 50 μm. **b** Western blot showed that FABP4 was increased in UUO mice. Schematic representation of quantitative data of indicated proteins. Representative images from three independent experiments are shown above. *n* = 6 mice. **c** Protein expression of FABP4 was detected in HK-2 cells treated with 10 ng/mL TGF-β for 48 h. Schematic representation of quantitative data of indicated proteins. Representative images from three independent experiments are shown above. **d** In the study of Ju CKD tubules, CKD was associated with significantly increased mRNA value of Fabp4 compared with biopsy samples from health control. **e** A negative correlation between tubular FABP4 and eGFR was observed in public Ju CKD dataset. Data were presented as mean ± SEM. *P < 0.05, **P < 0.01, ***P <0.001, ns means no statistical significance.

To investigate the role of FABP4 in fibrotic kidney, we first detected the expression of FABP4 in kidney TECs in UUO mouse models by immunofluorescence, we found that FABP4 was highly expressed in kidney tubular, and UUO injury caused increased FABP4 expression (Fig. 1a). As shown in Fig. 1b, c, in UUO mouse models and TGF-β-induced HK-2 cells, the expression of FABP4 was also significantly increased compared with control groups as well. Next, Nephroseq Analysis (https://www.nephroseq.org/resource /login.html) was conducted to evaluate the expression of Fabp4 in human kidney biopsy samples. In the study of Ju CKD tubules, CKD (eGFR less than 60 ml/min, *n* = 73) was associated with significantly increased mRNA value of Fabp4 compared with biopsy samples from health control (eGFR more than 90 ml/min, *n* = 63) (Fig. 1d). Moreover, a negative correlation between tubular FABP4 and eGFR was observed in the public Ju CKD dataset (*r*^*2*^ = 0.03704, *p* = 0.0085, *n* = 243) (Fig. 1e), suggesting a potential role of FABP4 in the development of CKD. These data show that the expression of FABP4 might be closely associated with CKD progression.

### Pharmacological inhibition of FABP4 by BMS309403 inhibits lipid accumulation in TECs during kidney fibrosis

**Fig. 2:**
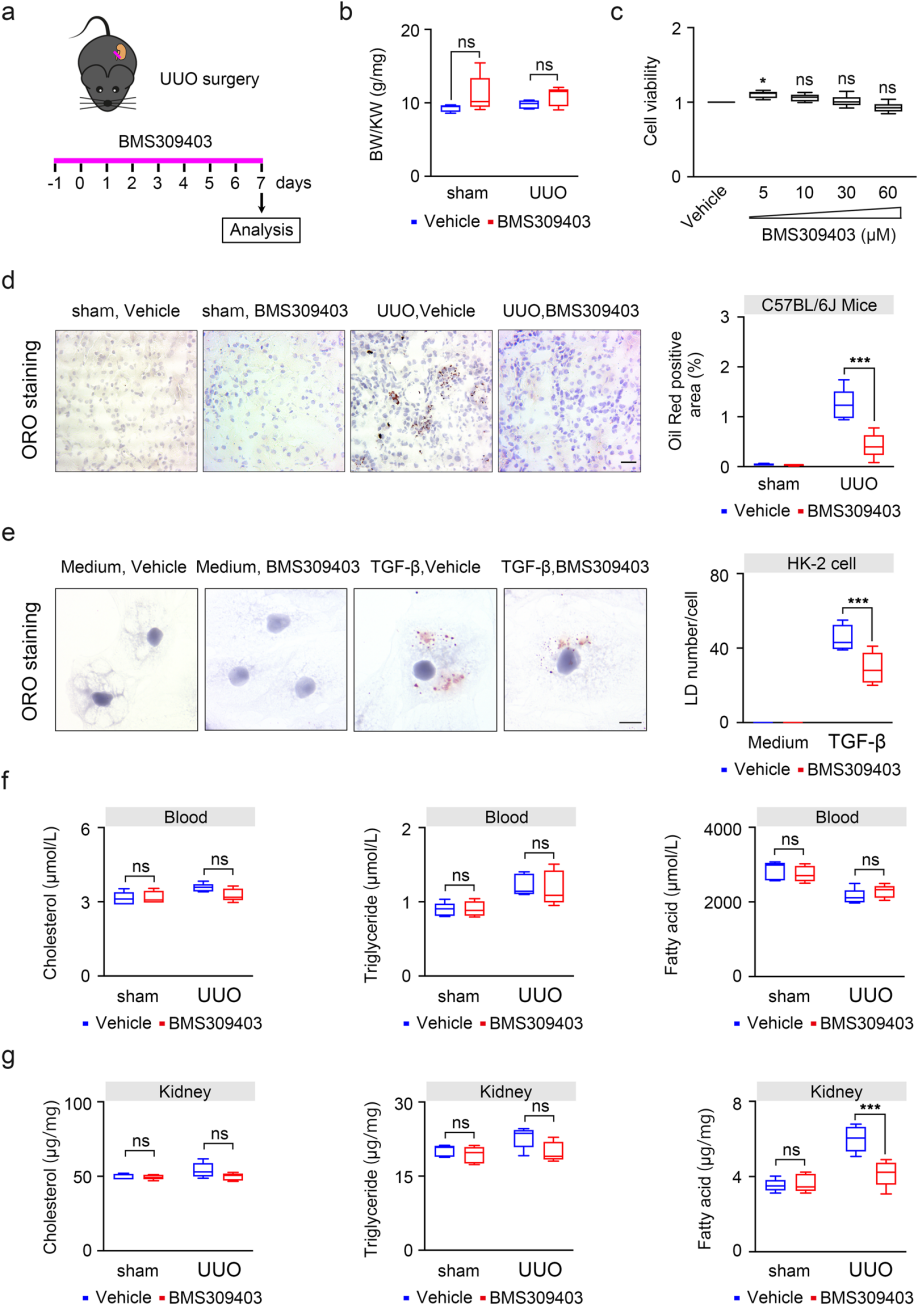
BMS309403 reduced lipid accumulation in TECs in vivo and in vitro. **a** One day before UUO surgery treatment with FABP4 inhibitor BMS309403, and then sacrificed on day 7. **b** BMS309403 has no significant change in the ratio of body weight to kidney weight (BW/KW). **c** Cell viability of HK-2 cells was detected by CCK8, which was treated by various doses of BMS309403 for 48 h. **d** Oil red O staining in frozen section from mice tubulointerstititum. **e** Oil red O staining in HK-2 cells co-treated with or without BMS309403 for 48 h. **f** Content of lipid in peripheral blood of mice. **g** Content of lipid in kidney tissue of mice. Data were presented as mean ± SEM. *P < 0.05, ***P < 0.001, ns means no statistical significance. Scale bars: 50 μm.

To evaluate the effect of FABP4 inhibitor BMS309403 in fibrotic kidney, we used UUO mouse models treated with or without BMS309403 and sacrificed on day 7 (Fig. 2a). As shown in Fig. 2b, there was no significant change in the ratio of body weight to kidney weight (BW/KW), indicating that BMS309403 had no significant adverse effects on the body fat (Fig. 2b). In HK-2 cells, CCK8 assay showed that BMS309403 did not lead to massive cell death after 48 h in a dose-dependent manner, which also indicated that BMS309403 has no adverse effect on cell viability (Fig. 2c). In pathological states, the accumulation of lipid droplets is considered as a marker for increased content of toxic lipid metabolites and dysregulation of fatty acid metabolism. The results of Oil Red O staining showed that the accumulation of lipid droplets in UUO-induced kidney and TGF-β treated HK-2 cells was obviously reduced after BMS309403 treatment (Fig. 2d, e). Lipid content in peripheral blood and kidney tissue was also detected to elucidate the effect of BMS309403 on lipid metabolism during kidney fibrosis. Interestingly, we found fatty acid content was only reduced in kidney tissue after treatment with BMS309403 (Fig. 2f). There was no significant change in TGs and cholesterol in the blood or kidney tissue treated with or without BMS309403 (Fig. 2f, g). These data indicate that BMS309403 may regulate lipid metabolism and mainly act on fatty acid in TECs.

### BMS309403 reduces fibrotic marker by decreasing the release of profibrotic cytokines in HK-2 cells

**Fig. 3:**
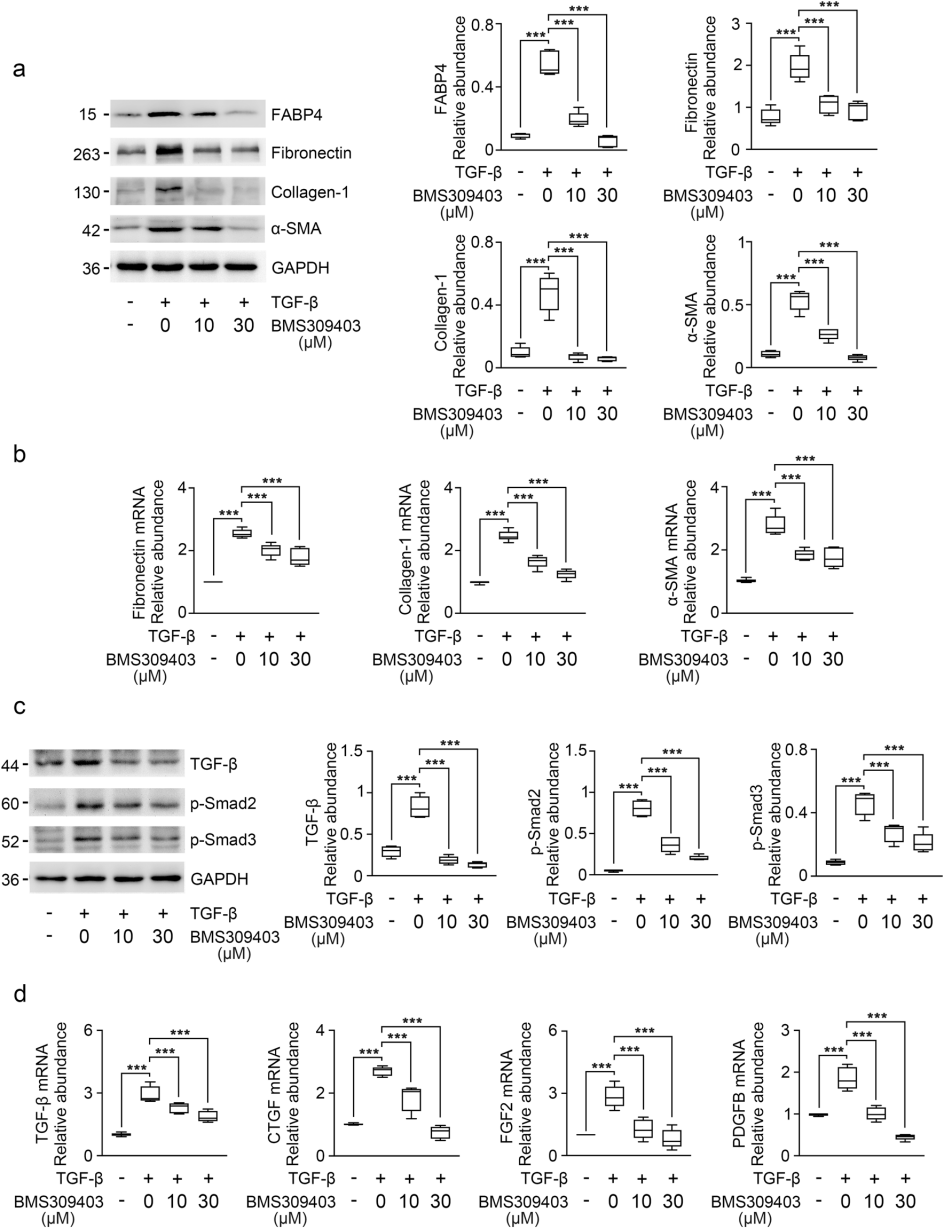
BMS309403 decreased the expression of fibrosis marker in TGF-β-treated HK-2 cells via inhibiting TGF-β/Smad pathway. **a** Western blot of HK-2 cell lysates for FABP4, fibronectin, collagen-I, and α-SMA expression. GAPDH sets as loading control. **b** RT-qPCR for fibronectin, collagen-I, and α-SMA transcripts normalized to Gapdh in HK-2 cells. **c** Western blot and band intensity quantitation for TGF-β, p-Smad2, and p-Smad3 in HK-2 cells. Blots were stripped and reprobed for GAPDH. **d** RT-qPCR for TGF-β, CTGF, FGF2, PDGFB transcripts normalized to Gapdh in total RNA extracts of HK-2 cells from DMSO- or BMS309403-treated normal and TGF-β-treated cells. These data were calculated from three independent experiments. ***P < 0.001.

To study the role of BMS309403 in TGF-β stimulated HK-2 cells, we determined the expression of FABP4 using immunoblotting. As shown in Fig. 3a, the protein level expression of FABP4 was remarkedly upregulated in TGF-β stimulated HK-2 cells. Further study showed that BMS309403 effectively reduced the protein and mRNA levels of fibronectin, collagen-I, and α-SMA in TGF-β stimulated HK-2 cells (Fig. 3a, b).

To investigate whether BMS309403 protected HK-2 cells from damage via inhibiting TGF-β/Smad signaling pathway, the protein expression levels of TGF-β, p-Smad2, and p-Smad3 were detected by immunoblotting. As shown in Fig. 3c, inhibition of FABP4 downregulated the protein levels of TGF-β and p-Smad3, as well as p-Smad2 in TGF-β treated HK-2 cells (Fig. 3c). BMS309403 also attenuated the release of various profibrotic cytokines, such as TGF-β, connective tissue growth factor (CTGF), fibroblast growth factor 2 (FGF2), and platelet-derived growth factor subunit B (PDGFB) in TECs (Fig. 3d). These data indicate that BMS309403 may prevent kidney fibrosis via inhibiting TGF-β/Smad signaling pathway and reducing the release of profibrotic cytokines from TECs.

### BMS309403 restores impaired FAO, attenuates apoptosis and ER stress in TGF-β stimulated HK-2 cells

**Fig. 4:**
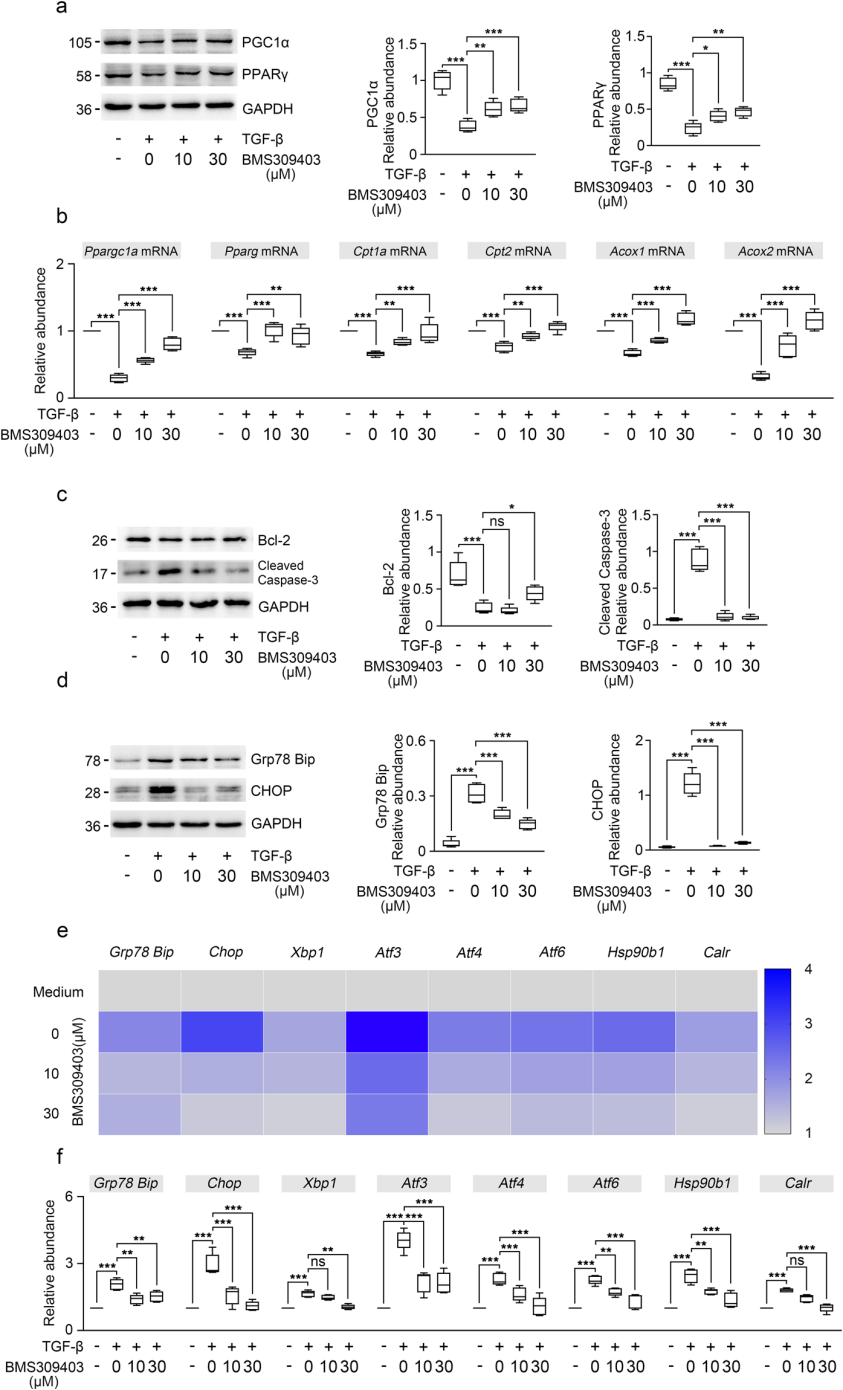
BMS309403 restored FAO enzymes activity, decreased apoptosis and ER stress in HK-2 cells. **a** The changes in PGC1α and PPARγ expression levels were confirmed in HK-2 cells by immunoblotting studies. **b** RT-qPCR for Ppargc1a, Pparg, Cpt1a, Cpt2, Acox1, and Acox2 transcripts normalized to Gapdh in HK-2 cells. **c** Western blot of whole-cell lysates for Bcl-2, cleaved caspase-3 expression in HK-2 cells. GAPDH sets as loading control. **d** Western blot of whole-cell lysates for Grp78 (Bip), CHOP expression in HK-2 cells. GAPDH sets as loading control. **e**, **f** ER stress related genes were analyzed by RT-qPCR in HK-2 cells from DMSO- or BMS309403-treated normal and TGF-β-treated cells. The transcript normalized to Gapdh. These data were calculated from three independent experiments. *P < 0.05, **P < 0.01, ***P < 0.001, ns means no statistical significance.

It has been reported that FAO deficiency plays a vital role in the pathogenesis of kidney fibrosis and restoring impaired FAO could be an effective strategy to alleviate kidney fibrosis^2,29^. To further explore the influence of BMS309403 on FAO activity in TGF-β stimulated HK-2 cells, we detected the key enzymes and transcription factors of FAO by immunoblotting. The results showed that the protein levels of PPARγ and PGC1α were obviously increased after BMS309403 treatment in TGF-β stimulated HK-2 cells (Fig. 4a). Consistent with immunoblot analysis, gene expression analysis also revealed the decreased level of Pparg, Ppargc1a, Cpt1a, Cpt2, Acox1, and Acox2 mRNA were partially recovered after BMS309403 treatment in TGF-β stimulated HK-2 cells compared to those in TGF-β treatment groups (Fig. 4b). These data suggest that FABP4 drives lipid accumulation and that it does so by regulating metabolism pathway.

Cell apoptosis and ER stress have been suggested to contribute to the development of kidney interstitial fibrosis^30–32^. Therefore, we determined whether BMS309403 can reduce kidney TECs apoptosis and ER stress in TGF-β stimulated HK-2 cells. As in vitro experiments, we found that apoptosis was relieved in the TGF-β stimulated HK-2 cells after BMS309403 treatment (Fig. 4c).

We evaluated the levels of ER stress in HK-2 cells by examining the expression of two unfolded protein response (UPR) molecules, Grp78 (Bip) and CHOP. As shown in Fig. 4d, BMS309403 mitigated the protein levels of Bip and CHOP in TGF-β stimulated HK-2 cells (Fig. 4d). We further examined the mRNA level of genes related to ER stress, including Bip, Chop, Xbp1, Atf3, Atf4 Atf6, Hsp90b1, and Calr, we found that these genes related to ER stress were all elevated after TGF-β treatment, indicated activated ER stress during kidney fibrosis. However, these abovementioned genes could be further alleviated by BMS309403 administration (Fig. 4e, f). These results indicate that BMS309403 ameliorates kidney fibrosis by reducing TECs apoptosis and inhibiting ER stress.

### BMS309403 prevents kidney injury in the fibrotic kidney

**Fig. 5:**
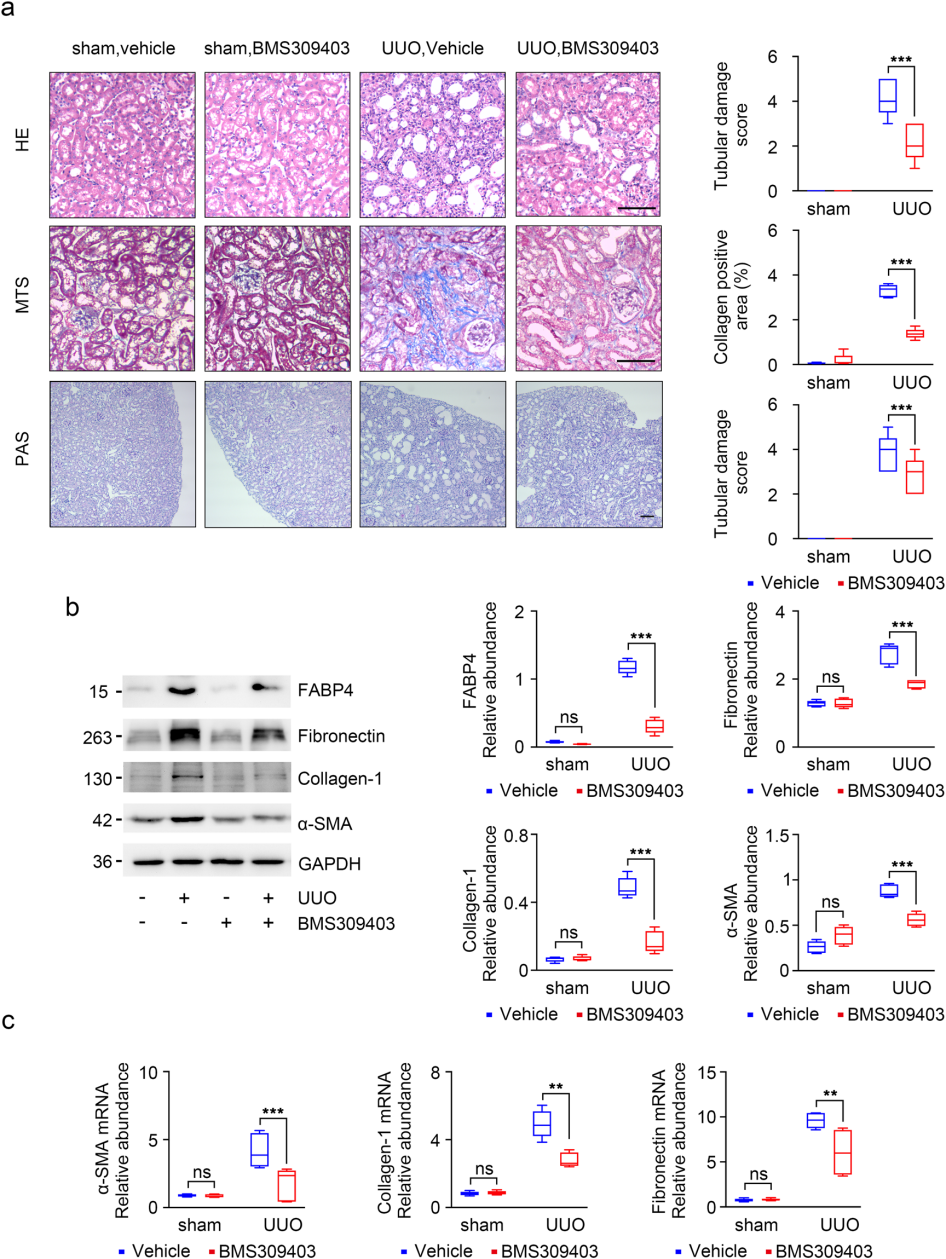
BMS309403 improved kidney lesion by UUO. **a** Staining with HE, PAS, and MTS of kidney sections from obstructed (UUO) or sham-operated kidneys (control). Animals received BMS309403 (40 mg/kg for 8 days) or vehicle as indicated. *n* = 6 per group. **b** Western blot of whole-kidney lysates for FABP4, fibronectin, collagen-I, and α-SMA expression. GAPDH sets as loading control. **c** Fibronectin, collagen-I, and α-SMA mRNA expression by RT-qPCR. Schematic representation of quantitative data of indicated proteins. The transcripts normalized to Gapdh. Representative images from three independent experiments are shown above. **P < 0.01, ***P < 0.001, ns means no statistical significance. Scale bars: 25 μm.

To further investigate the role of BMS309403 in kidney injury and fibrosis, BMS309403 was administered by gavage about 1 h before surgery and daily during UUO operation. The kidney morphological changes in UUO groups included degeneration of tubular epithelia with loss of brush borders and dilatation (Fig. 5a). These tubular changes were considerably attenuated by the administration of BMS309403 (Fig. 5a). PAS staining also revealed tubular epithelial disruption with sloughing off of the epithelia and shedding of PAS-positive material in the tubular lumina (Fig. 5a). Interestingly, these changes were largely reversed in UUO mice that received prior treatment with BMS309403 (Fig. 5a). MTS further verified that BMS309403 attenuated collagen deposition in kidney interstitium in UUO mice (Fig. 5a). Of note, western blot indicated that FABP4 was significantly elevated in UUO group than controls, and was markedly attenuated after BMS309403 treatment (Fig. 5b). Consistent with the reduction of MTS of fibrosis in kidneys of anti-FABP4-treated animals, the increasing of fibrotic markers (fibronectin, collagen-I, and α-SMA), known to occur in UUO mouse models, were markedly reduced specifically by BMS309403 treatment by Western blot (Fig. 5b). It was consistent with the mRNA levels of fibronectin, collagen-I, and α-SMA (Fig. 5c). These observations suggest that FABP4 inhibitor reduce interstitial collagen deposition and tubule lesion, may provide new evidence for the treatment of CKD.

### BMS309403 decreases the release of profibrotic cytokines and improves FAO impaired in TECs in UUO mice

**Fig. 6:**
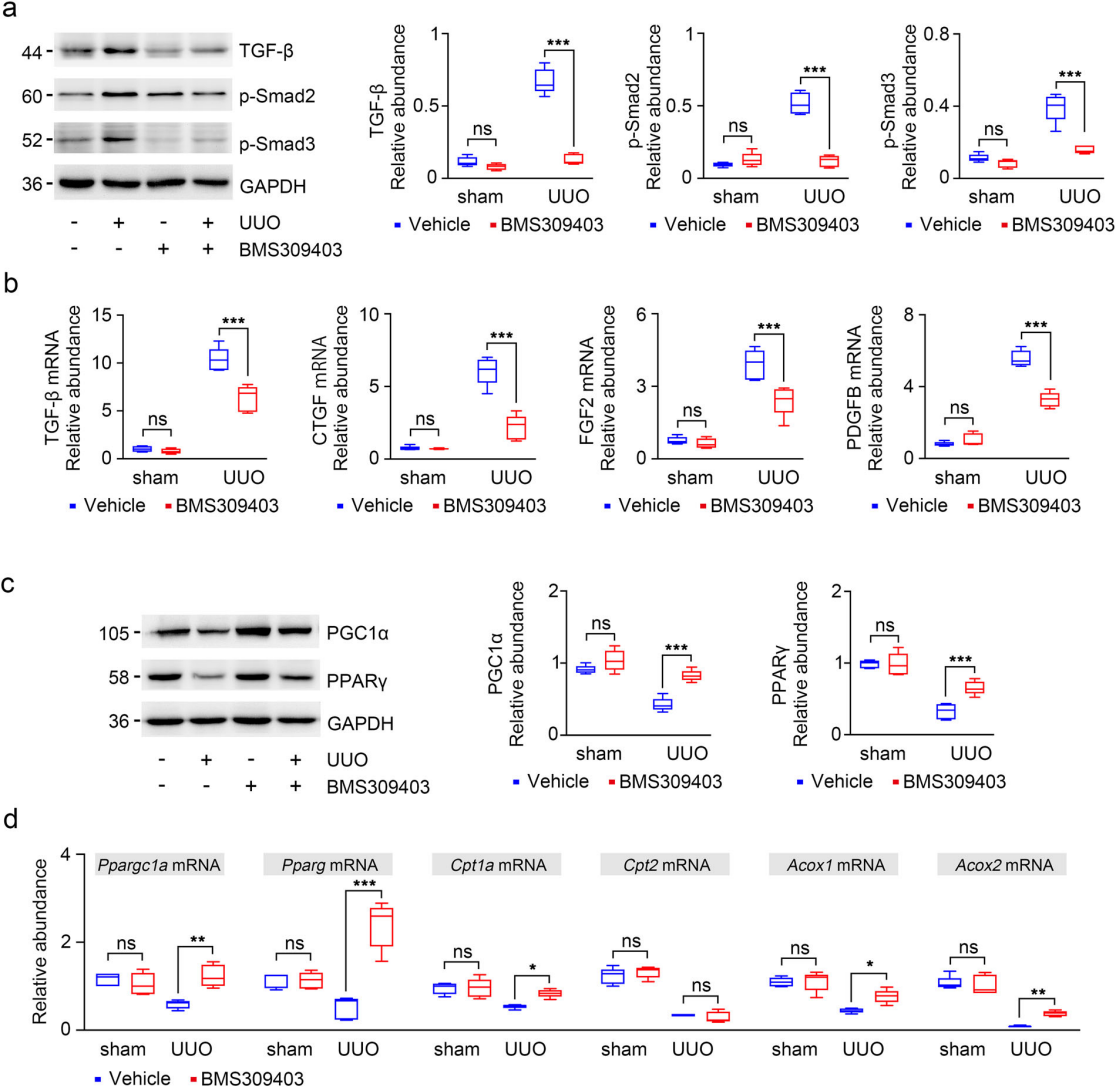
BMS309403 reduced the release of profibrotic cytokines by inhibiting TGF-β/Smad3 pathway and restored impaired FAO pathway in fibrotic kidney. **a** TGF-β, p-Smad2, and p-Smad3 expression was verified in kidney samples by Western blot. Blots were stripped and reprobed for GAPDH. **b** RT-qPCR for TGF-β, CTGF, PDGFB, and FGF2 transcripts normalized to Gapdh in kidney tissue. **c** The changes in PGC1α and PPARγ expression levels were confirmed in mouse kidneys by immunoblotting studies. **d** Ppargc1a, Pparg, Cpt1a, Cpt2, Acox1, and Acox2 mRNA expression were analyzed by RT-qPCR in total RNA extracts of kidneys from saline- or BMS309403-treated normal and UUO mice. The transcript normalized to Gapdh. Representative images from three independent experiments are shown above. *P < 0.05, **P < 0.01, ***P < 0.001, ns means no statistical significance.

To investigate the mechanism by which FABP4 amplifies injury and fibrosis in the damaged kidney, we detected the expression of TGF-β/Smad signal pathway by immunoblotting. As shown in Fig. 6a, inhibition of FABP4 downregulated the protein levels of TGF-β and p-Smad3, as well as p-Smad2 in both UUO engaged kidney (Fig. 6a). BMS309403 also attenuated the release of various profibrotic cytokines (TGF-β, CTGF, FGF2, and PDGFB) in TECs (Fig. 6b). These data indicate that BMS309403 may prevent kidney fibrosis via inhibiting TGF-β/Smad signaling pathway and reduce the release of profibrotic factors by TECs.

We further determined the effect of FABP4 on metabolic pathway, specifically the FAO pathway in vivo. We found that the protein levels of PPARγ and PGC1α were decreased in fibrotic kidney, and upregulated after BMS309403 treatment (Fig. 6c). Furthermore, BMS309403 restored defective FAO in kidney injury, it upregulated a series of FAO-related enzyme transcripts (Pparg, Ppargc1a, Cpt1a, Cpt2, Acox1, and Acox2) (Fig. 6d). These results suggest that BMS309403 protects kidney from lipotoxicity through increasing β-oxidation of TECs.

### FABP4 inhibitor attenuates TECs apoptosis and ER stress in UUO mouse model

**Fig. 7:**
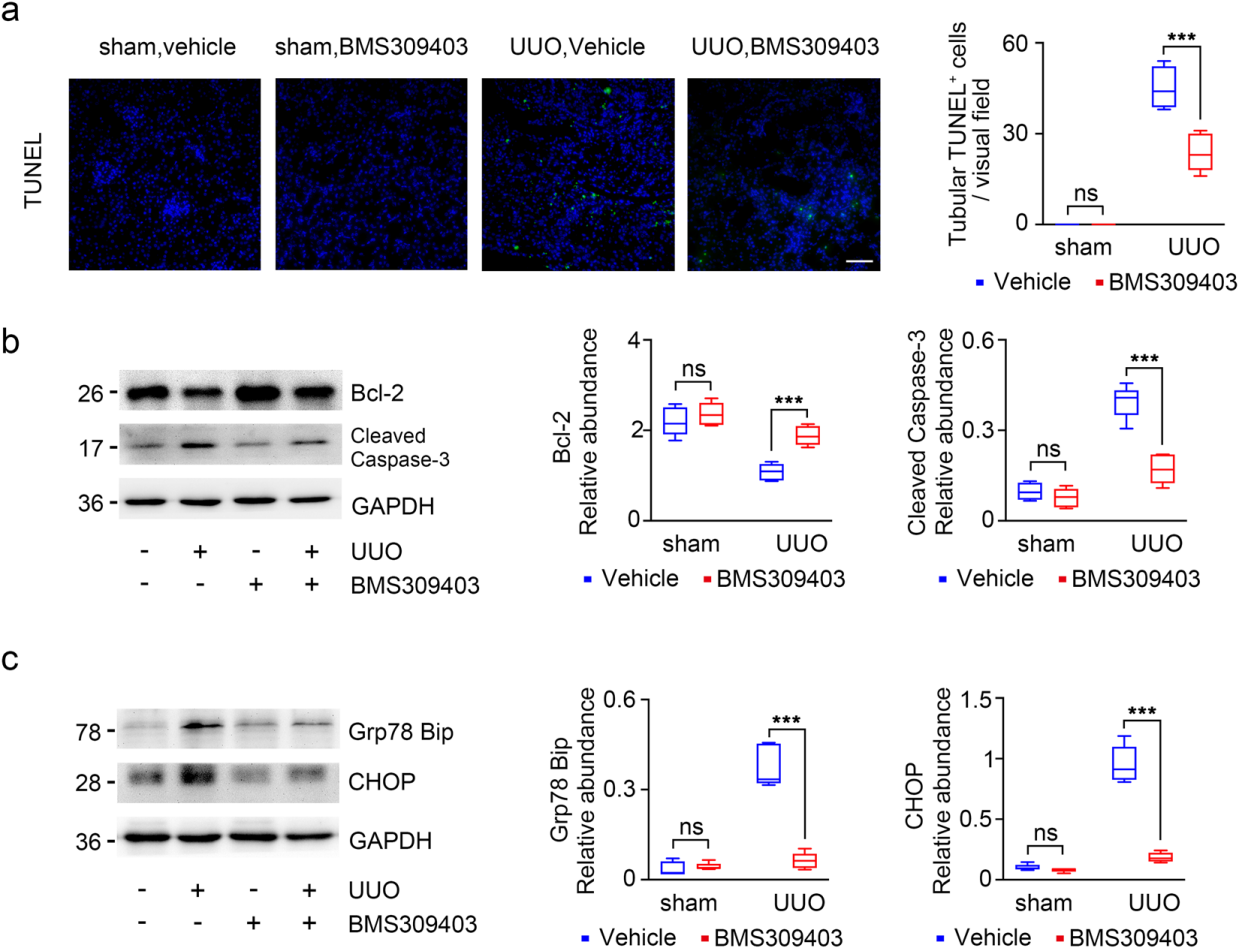
Apoptosis and ER stress were significantly ameliorated in UUO kidneys after BMS309403 treatment. **a** TUNEL staining of kidney tissues (green: TUNEL-positive cells). **b** Western blot of whole-kidney lysates for Bcl-2, cleaved caspase-3 expression in mice. GAPDH sets as loading control. **c** Western blot of whole-kidney lysates for Grp78 (Bip), CHOP expression in mice. GAPDH sets as loading control. Representative images from three independent experiments are shown above. ***P < 0.001, ns means no statistical significance. Scale bars: 50 μm.

Tubular cell apoptosis was evaluated by TUNEL staining, the result showed that TUNEL-positive cells were rarely observed in UUO mice after BMS309403 treatment (Fig. 7a). As shown in Fig. 7b, the result indicated that BMS309403 suppressed the expression of the apoptotic protein caspase-3 in UUO mice, and upregulated the expression of the anti-apoptotic protein Bcl-2 (Fig. 7b). Furthermore, tissue sections from UUO injured kidneys showed significantly higher levels of ER stress, BMS309403 treatment protected UUO mice against ER stress (Fig. 7c). These data suggest that BMS309403 may treat CKD via mitigating TECs apoptosis and excessive ER stress.

## Discussion

Numerous work has already demonstrated the promising effect of FABP4 inhibition in preventing obesity, atherosclerosis, diabetes mellitus, metabolic syndrome, and AKI^19,20,24^, although recent work reported that FABP4 knockout mice improve kidney function and attenuate fibrotic response during kidney fibrosis^22,23^, the mechanistic function of pharmacological FABP4 inhibition has not yet been determined. In the present study, we reported the preemptive pharmacological effect of FABP4 inhibition both in UUO-induced fibrotic kidney in vivo and TGF-β initiated tubular cells injury in vitro. Selective inhibition of FABP4 by BMS309403 significantly attenuated tubulointerstitial damage and matrix collagen deposition, the expression of fibrotic markers: fibronectin, α-SMA, and collagen-1, as well as the tubular lipid deposition during kidney fibrosis. Mechanistically, BMS309403 treatment remarkably improves the tubular cells FAO, and lipotoxicity such as apoptosis and ER stress both in UUO mouse model and in TGF-β stimulated TECs. Together, these data indicate that the renoprotective role of FABP4 inhibition against kidney fibrosis, and is at least partially thought of mediating metabolic activity.

FABP4 is a member of FABP family that has been recognized as a lipid-binding chaperon for long-chain unesterified fatty acids and involves body weight control, glucose metabolism, lipid metabolism, and insulin resistance^33^. In the normal kidney, FABP4 is detected in peritubular capillary endothelial cells in both cortex and medulla^18^. Recently, studies have shown that FABP4 contributes to the pathogenesis of a variety of kidney diseases^19–23^. It is reported that plasma FABP4 concentrations are directly associated with serum creatinine and an inverse correlation between FABP4 levels and eGFR in type 2 diabetic patients independent of microalbuminuria^34^. Later studies further demonstrate urinary FABP4 level is associated with progression of proteinuria and decline of eGFR^34^. In a bidirectional cross-sectional study, Tsai et al show that urinary level of FABP4 could be a novel predictor for the CKD progression in severe NAFLD patients with hypertension^35^. Moreover, Fabp4 knockout mice have significantly reduced the apoptosis of kidney tubules, and alleviated kidney lesions in UUO mouse models, indicating that the expression of FABP4 is closely bound up with kidney injury^22,23^. Consistent with these findings, our study has determined a critical role of FABP4 inhibition in preventing kidney fibrosis by pharmacological approach, which strongly indicates FABP4 might be a potential target against kidney fibrosis.

During kidney fibrosis, the profibrotic cytokines can be produced by injured TECs and contribute to the pathogenesis of fibrotic diseases. In our study, four well-documented profibrotic cytokines, including TGF-β, CTGF, FGF2, and PDGFB, which could promote fibroblast proliferation, collagen deposition, and lead to kidney fibrosis, were examined^36^. We found that inhibition of FABP4 by BMS309403 alleviated kidney lesion and interstitial fibrosis by inhibiting the TGF-β/Smad signaling pathway, as well as decreased expression of varieties of profibrotic cytokines such as CTGF, FGF2, and PDGFB. This is an interesting found in our study, although the precise mechanism is still unknown. Taking into account the role of FABP4 inhibitor in regulating lipid metabolism, we speculate that coordinated impaired FAO activities and lipotoxicity occurred in tubular cells during kidney fibrosis to assist the production of profibrotic secretory proteins.

Kidney TECs are highly dependent on FAO to generate large amounts of ATP to perform normal physiological activity^2^. More and more evidence has highlighted that dysregulation of cellular metabolism is closely related to kidney fibrosis. In DN, the expression of fatty acid synthase, including sterol regulatory element-binding protein 1c (SREBP1c) and carbohydrate-responsive element-binding protein (CHREBP), are elevated^37^. Additionally, activation of 5′-AMP-activated protein kinase (AMPK) using metformin inhibits the synthesis of cholesterol, TGs, and fatty acids through phosphorylating acetyl-CoA carboxylase 1 (ACC1) and SREBP1c, thereby mitigating fibrosis^37^. Kang et al have indicated that levels of key regulators of FAO are lower in CKD samples compared to control samples by gene ontology analysis; in various mouse models of kidney fibrosis, such as diabetic kidney injury induced by streptozotocin and folic acid-induced fibrosis, both found that fatty acid utilization is reduced, levels of key and limiting enzymes are markedly lower than controls^2^. PPARγ is considered to be the target gene by FABP4, PPARγ regulates the progress of fatty acid transport, oxidation, and decomposition by regulating the expression levels of fatty acid transporter, FABP, and carnitine palmitoyltransferase-1 (CPT1)^38^. Consistent with our study, we found that inhibition of FABP4 upregulated the expression of PPARγ and the multiple FAO-related target genes downstream including CPT1, CPT2, and ACOXs, restored the FAO ability of kidney TECs, and attenuated abnormal lipid accumulation caused by fibrotic kidney.

ER is essential for normal physiological function in cells with a high rate of protein and lipid synthesis^39^. Pathophysiological states that overwhelm the capacity for protein folding or that disrupt normal folding processes result in the accumulation of misfolded proteins in the ER and dilatation of the ER, leading to ER stress and activation of the UPR^40–42^. Moderate ER stress could alleviate the damage caused by stress, but prolonged or severe stress response leads to apoptosis and may thus be an important factor in the pathogenesis of many diseases. Abundant evidence has indicated that ER stress is tightly associated with the development of glomerular and tubular disease including DN, AKI, and CKD^32,43^. It has been shown that UPR sensors directly engaged in the regulation of lipid metabolism, and lipotoxicity can activate an ER stress response in the liver. Therefore, we assumed that BMS309403 might inhibit ER stress to improve kidney fibrosis via directly regulating lipid homeostasis.

## Conclusion

**Fig. 8:**
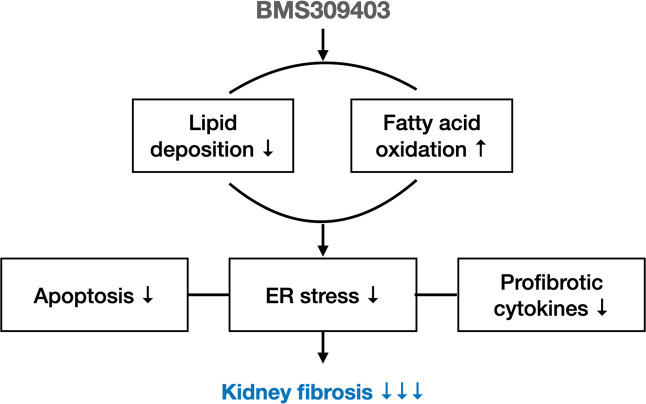
Schematic diagram of the mechanisms of FABP4 in kidney fibrosis. BMS309403 reduces lipotoxicity to improve kidney fibrosis mainly by reducing lipid accumulation and increasing FAO pathway, and also reduce the secretion of a series of profibrotic cytokines by kidney TECs to relieve kidney fibrosis.

In summary, our present findings reveal that preemptive chemical inhibition of FABP4 may show beneficial effects against fibrotic kidneys and unravel a mechanism by which FABP4 inhibitor could mediate its anti-fibrotic effects through modulating tubular lipid metabolism (Fig. 8). Thus, our findings offer tools to slow the progress of kidney fibrosis that may facilitate therapeutic applications including that of FABP4 inhibition.

## Supplementary information

supplementary data

## Data Availability

The data that support the findings of this study are available from the corresponding author upon reasonable request.
