# Supplementary material for: Pre-emptive pharmacological inhibition of fatty acid–binding protein 4 attenuates kidney fibrosis by reprogramming tubular lipid metabolism
Source: Cell Death Dis. 2021 Jun 3;12(6):572. doi: 10.1038/s41419-021-03850-1 (PMC8175732; doi:10.1038/s41419-021-03850-1)
Supplement: Supplementary file 1 — supplementary data [file 41419_2021_3850_MOESM1_ESM.docx]

**Supplementary Date**

**Table 1**

Capital letters refer to human genes and lowercase letters represent mouse genes.

| Gene | Forward (5’→3’) | Reverse (5’→3’) |
| --- | --- | --- |
| FN | AATAGATGCAACGATCAGGACA | GCAGGTTTCCTCGATTATCCTT |
| COL1A1 | AAAGATGGACTCAACGGTCTC | CATCGTGAGCCTTCTCTTGAG |
| ACTA2 | CTCTGGACGCACAACTGGCATC | GGCATGGGGCAAGGCATAGC |
| TGFB1 | CTGTACATTGACTTCCGCAAG | TGTCCAGGCTCCAAATGTAG |
| FGF2 | CATCAAGCTACAACTTCAAGCA | CCGTAACACATTTAGAAGCCAG |
| PDGFB | GATCCGCTCCTTTGATGATCTC | GGTCATGTTCAGGTCCAACTC |
| CTGF | ATTCTGTGGAGTATGTACCGAC | GTCTCCGTACATCTTCCTGTAG |
| PGC1A | CAGAGAGTATGAGAAGCGAGAG | AGCATCACAGGTATAACGGTAG |
| PPARG | AGATCATTTACACAATGCTGGC | TAAAGTCACCAAAAGGCTTTCG |
| CPT1A | GATTTCCATTCCTTCCCATTCG | CTCGTATGTGAGGCAAAACTTG |
| CPT2 | GCCTAGATGACTTCCCCATTAA | AAAGGATTTATCAAACCAGCGG |
| ACOX1 | CACAAGTAAACCAGCGTGTAAA | GTTCTTAGCCCACTCAAACAAG |
| ACOX2 | ACTGAAGCCACCTATGACGC | AGTCTCCAGGCCACCATTTG |
| CHOP | GAGAATGAAAGGAAAGTGGCAC | ATTCACCATTCGGTCAATCAGA |
| BIP | CAGTTGTTACTGTACCAGCCTA | CATTTAGGCCAGCAATAGTTCC |
| ATF3 | TAGCCCCTGAAGAAGATGAAAG | CTTCTTCTTGTTTCGGCACTTT |
| ATF4 | ATGGATTTGAAGGAGTTCGACT | AGAGATCACAAGTGTCATCCAA |
| XBP1 | CTTGTAGTTGAGAACCAGGAGT | CCCAACAGGATATCAGACTCTG |
| HSP90B1 | TCTGAATTGATTGGCCAGTTTG | GGGTATCGTTGTTGTGTTTTGA |
| CALR | AGATAAAGGTTTGCAGACAAGC | CATGTCTGTCTGGTCCAAACTA |
| GAPDH | ACCAAATCCGTTGACTCCGAC | CTCCTGTTCGACAGTCAGCC |
| Fabp4 | CATCCGGTCAGAGAGTACTTTT | TAGGGTTATGATGCTCTTCACC |
| Fn | ACAGTCCAGCAAGCAGCAAGC | TGGTGGTCACTCTGTAGCCTGTC |
| Col1a1 | GGCAAAGATGGAGAAGCTGG | GGAAACCTCTCTCGCCTCTT |
| Acta2 | GGCTTCGCTGGTGATGATGCTC | TCCCTCTCTTGCTCTGGGCTTC |
| Tgfb1 | CCAGATCCTGTCCAAACTAAGG | CTCTTTAGCATAGTAGTCCGCT |
| Fgf2 | AGTTGTGTCTATCAAGGGAGTG | CATTGGAAGAAACAGTATGGCC |
| Pdgfb | GTCCAGGTGAGAAAGATTGAGA | GTCATGGGTGTGCTTAAACTTT |
| Ctgf | AAAGCAGCTGCAAATACCAATG | AAATGTGTCTTCCAGTCGGTAG |
| Pgc1a | GGATATACTTTACGCAGGTCGA | CGTCTGAGTTGGTATCTAGGTC |
| Pparγ | CCAAGAATACCAAAGTGCGATC | TCACAAGCATGAACTCCATAGT |
| Cpt1 | CTACATCACCCCAACCCATATT | GATCCCAGAAGACGAATAGGTT |
| Cpt2 | TGTCTTTGATGTCCTCGATCAA | TCGGTTCTCACTGGTCAAATAA |
| Acox1 | CCAATGCTGGTATCGAAGAATG | CGACTGAACCTGGTCATAGATT |
| Acox2 | CAATGACTTCCATCAAGTGGTG | GTCTATGTTTTCGAAGCCCATC |
| Gapdh | AATGGTGAAGGTCGGTGT | GTGGAGTCATACTGGAACATGTAG |
